# Innovation in Evaluating the Impact of Integrated Service-Delivery: The Integra Indexes of HIV and Reproductive Health Integration

**DOI:** 10.1371/journal.pone.0146694

**Published:** 2016-01-22

**Authors:** Susannah H. Mayhew, George B. Ploubidis, Andy Sloggett, Kathryn Church, Carol D. Obure, Isolde Birdthistle, Sedona Sweeney, Charlotte E. Warren, Charlotte Watts, Anna Vassall, Integra Initiative

**Affiliations:** 1 Department of Global Health & Development, London School of Hygiene & Tropical Medicine, 15–17 Tavistock Place, London, WC1H 9SH, United Kingdom; 2 Centre for Longitudinal Studies, Department of Social Science, UCL Institute of Education, University College London, 55–59 Gordon Square, London, WC1H 0NU, United Kingdom; 3 Department of Population Health, London School of Hygiene & Tropical Medicine, Keppel St, London, WC1E 7HT, United Kingdom; 4 Department of Epidemiology and Public Health, Swiss Tropical and Public Health Institute, P.O. Box 4002, Basel, Switzerland; 5 Reproductive Health Services and Research, Population Council, Suite 280, 4301 Connecticut Ave NW, Washington, District of Columbia, 20008, United States of America; Vanderbilt University, UNITED STATES

## Abstract

**Background:**

The body of knowledge on evaluating complex interventions for integrated healthcare lacks both common definitions of ‘integrated service delivery’ and standard measures of impact. Using multiple data sources in combination with statistical modelling the aim of this study is to develop a measure of HIV-reproductive health (HIV-RH) service integration that can be used to assess the degree of service integration, and the degree to which integration may have health benefits to clients, or reduce service costs.

**Methods and Findings:**

Data were drawn from the Integra Initiative’s client flow (8,263 clients in Swaziland and 25,539 in Kenya) and costing tools implemented between 2008–2012 in 40 clinics providing RH services in Kenya and Swaziland. We used latent variable measurement models to derive dimensions of HIV-RH integration using these data, which quantified the extent and type of integration between HIV and RH services in Kenya and Swaziland. The modelling produced two clear and uncorrelated dimensions of integration at facility level leading to the development of two sub-indexes: a Structural Integration Index (integrated physical and human resource infrastructure) and a Functional Integration Index (integrated delivery of services to clients). The findings highlight the importance of multi-dimensional assessments of integration, suggesting that structural integration is not sufficient to achieve the integrated delivery of care to clients—i.e. “functional integration”.

**Conclusions:**

These Indexes are an important methodological contribution for evaluating complex multi-service interventions. They help address the need to broaden traditional evaluations of integrated HIV-RH care through the incorporation of a functional integration measure, to avoid misleading conclusions on its ‘impact’ on health outcomes. This is particularly important for decision-makers seeking to promote integration in resource constrained environments.

## Introduction

Since the 1990s there has been an assumption that integration of HIV-related services (e.g. HIV testing, condom provision, and HIV treatment) with family planning (FP), antenatal care (ANC) and postnatal care (PNC) services, would streamline service delivery. In practice, countries take myriad different approaches to the organisation of care, and ‘integrated’ primary care services remain poorly defined.[1–3] In lower- and middle-income contexts it is usually understood as the amalgamation of previously separate components of care, or the addition of a new intervention into an existing service (e.g. adding HIV testing to FP services).[4] In industrialised country settings it is often interpreted as a mechanism to improve the coordination of care between different organisations and professional bodies at different levels of the health system.[5] As a result, there is no standard definition of “integration” which has been variously categorised by different authors and studies.[4, 6–10]

The multi-dimensional nature of integration raises the fundamental question of how ‘integration’ or improvements in integration should be measured. Without clarity on this, it is difficult to assess the extent to which services are integrated, and whether the delivery of integrated services leads to cost savings, greater client satisfaction, and/or improved patient outcomes. To date, only a few studies have attempted to measure, rank, or assess different levels or types of HIV/reproductive health (RH) service integration.[11] One study in the US attempted to rank clinics by their degree of integration of HIV with primary care services, determined by interviews with facility managers, then applied this to HIV client visit data to give an ‘index of integrated care utilisation’.[12] Another in South Africa attempted to quantify different levels of integration, again based on questionnaires with staff.[13] A third sought to describe the relationship between integration and ‘teen-friendly’ service outcomes, and measured integration through staff and client interviews.[14]

The Integra Initiative is the largest complex evaluation of its kind seeking to determine the impact of service integration on service and health outcomes in Kenya and Swaziland. Integra’s focus is the integration of HIV/sexually transmitted infection (HIV/STI) services (including HIV/STI counselling, testing and treatment) and reproductive health (RH) services (including: FP, ANC and PNC), for which multiple benefits have been claimed yet the evidence is at best inconsistent.[15, 16] Integra’s initial intent was to conduct a pre/post study with pair-matched intervention (integrated) and comparison (non-integrated) sites. However, as with many other pragmatic trials of this kind, the study had no direct control over clinic organisation, but instead supplied inputs to facilitate integration such as training and supplies. In such a ‘real world setting’ this lack of control, high staff turnover and migration between sites, external donor activities and evolving policy meant that over time some control sites also integrated services during the study. It therefore quickly became clear that any longitudinal analysis would be confounded by the varying levels of integration already existing prior to the start of the intervention and then developing over time, independently, in both intervention and comparison sites. To address this, a need was identified to develop a tool to capture and measure the extent of integration at the study sites, and then use this to explore the relationship between integration and a range of study outcomes.

This situation is not unique: when evaluating complex multi-service health interventions within ‘real world’ settings, study designs that capture the process and extent of the implementation of the intervention, both at baseline and throughout the course of the study are becoming increasingly necessary, particularly where organisational change is involved.[17–19] This paper thus aims to contribute to the field of complex intervention evaluation, as well as the broader policy debate on health service integration, by describing the development of a tool to measure the degree of HIV-RH integration achieved in the health facilities studied: the Integra Index.

## Methods

### The Integra Initiative

The Integra Initiative is a non-randomised, pre/post intervention trial using household and facility-based data. Integra uses mixed methods to analyse a hypothesised causal pathway between integration and its theorised outcomes, which include costs, quality of care, service utilisation, stigma and sexual and reproductive behaviours. Integra is embedded research, working in public-sector and NGO facilities in Kenya and Swaziland. It aims to evaluate the impact of different models of delivering integrated HIV-RH services in Kenya and Swaziland on a range of health and service outcomes.[20] Research was conducted in both high- and moderate-HIV prevalence settings (Swaziland, 10 clinics; and Kenya, 30 clinics). An intervention was developed in collaboration with the national Ministries of Health to support service integration in study clinics (see Warren et al. for details[20]). The 40 study facilities consisted of: primary care clinics including dispensaries, health centres, public health units (n = 30); and secondary out-patient clinics at district or provincial hospitals (n = 10). A total of 32 clinics were primarily managed by the public sector, while eight were run by an NGO (6 in Kenya, 2 in Swaziland).

#### Ethics

Ethical clearance was granted by the Kenya Medical Research Institute (KEMRI) Ethical Review Board (approval # KEMRI/RES/7/3/1, protocol #SCC/113 and #SCC/114), the Ethics Review Committee of the London School of Hygiene & Tropical Medicine (LSHTM) (#5426 and #5436) and the Population Council’s Institutional Review Board (#443 and #444). The Integra Initiative is a registered non-randomised trial: ClinicalTrials.gov Identifier: NCT01694862.

Data collected are described below, but involved either routine aggregated clinic data (on human and physical resource-use) or anonymised (at point of data collection) data from clients using the facility during the five-day client flow data collection period (about services received). No personal or client-identifying information was collected and data were only used in aggregate form per clinic. No individuals’ clinical records were used and no individual provider details were recorded. For providers who were interviewed, individual written informed consent was obtained, recorded on an informed-consent sheet. In addition, verbal informed consent was obtained from facility managers for all facilities involved in the Integra study. These forms of consent were approved by the above ethics boards.

### Measurement & ranking of integrated care: the Integra Index

The Integra Index was developed to measure the level of integration at baseline and follow-up in all the 40 study facilities. The construction of the Index involved four steps, detailed below.

#### 1) Identification of integration attributes and indicators

We reviewed the key aspects of integration identified in the literature [4–10, 21–23] to help identify the dimensions of integration that needed to be measured. The dimensions emerged as: 1) physical integration—the most common way for integration to be described, in terms of multiple services being available at the same facility (with or without referrals between units/rooms) or in the same room;[4, 6, 7, 22] 2) Temporal integration—multiple services being available throughout the week vs. on specific days;[8] 3) Provider-level integration—one provider gives multiple services in a consultation (either actively or in response to client demands) or over one day.[9, 22, 23] In addition, we identified a fourth, higher level dimension: receipt of integrated care by clients, or what we termed “Functional integration” within a single visit or consultation. We validated these dimensions by consulting with service providers and researchers from Kenya and Swaziland (described in 3) below).

As the index was developed post initial study design, we then assessed the study data we had to identify the best and most feasible measure of each integration dimension. The multi-disciplinary research team (including evaluation researchers, epidemiologists, health systems researchers, economists and statisticians) identified eight ‘attributes’ from our study data that best reflected the above dimensions of integration: physical (what rooms/buildings different services are delivered in); temporal (on what days/times); provider (by whom); and functional (defined as “actual services received by client”) (Table 1). Given the study’s focus, the indicators measured the provision/receipt of any RH service (FP, ANC, PNC) AND any HIV/STI service (HIV counselling and testing, HIV anti-retroviral therapy (ART) treatment, CD4 count services, STI treatment, cervical cancer screening).

**Table 1:** Index dimensions, attributes (indicators) and data source.

Dimension	Attribute and indicator description	Data source
Physical Integration	Service availability at MCH/FP* ***Unit*:** % of HIV-related services [1–5 below^†^] available in the MCH/FP unit at each facility.	Periodic Activity Review
	***Service availability at facility*:** % of RH [6–8 below^†^] and HIV-related services available anywhere in the facility	Periodic Activity Review
	***Range services per room*:** % HIV-related services that are provided in each MCH/FP consultation room	Costing study (registers)
	***HIV treatment location and referral***^‡^**:** location of ART and functionality of referral system to ART for SRH clients	Client Flow tool
Temporal integration	***Range of services accessed daily*:** % days in the week on which any RH services AND any HIV-related services are accessed	Client Flow tool
Provider Integration	***Range of services per provider*:** % HIV-related services that are provided per MCH/FP clinical staff member in a day	Costing Study (registers)
Functional Integration	***Range of services provided in one consultation*:** % clients who receive any RH services AND any HIV-related services in one of their provider contacts	Client flow tool
	***Range of services provided in one visit to facility*:** % who receive any RH services AND any HIV-related services during their visit to the facility (one day)	Client flow tool

*Maternal and child health/family planning unit

^†^ Range of services assessed: **HIV-related services are** 1) Antiretroviral therapy (ART); 2) Cervical cancer screening; 3) CD4 count services; 4) HIV/AIDS testing services; 5) STI treatment. **RH services are** 6) Family Planning; 7) Post-natal care; 8) Antenatal care

^‡^ We recognised that the appropriateness of including this indicator is dependent on the need for ART in the catchment population; we took into account the fact that smaller clinics do not provide ART on site by using a graded scoring system incorporating referrals, as follows. HIV treatment score**:** 0 = Received no ART ("HIV care") and not referred for ART; 1 = Referred for ART but not received during that visit; 2 = Received ART during visit, either as 1 service only, or as additional service but with a different provider; 3 = Received ART in addition to an SRH service (FP/ANC/PNC/STI) with the same provider.

#### 2) Use of clinic data to generate attribute scores

We drew on two Integra datasets to capture the eight selected attributes, with data from each collected from the 40 clinics at baseline (2008–9) and endline (late 2010-early 2012): (i) a dataset used for costing ‘the economics dataset’ (that included service statistics) and (ii) a client flow dataset. Summary descriptions are provided in Table 2.

**Table 2:** Description of Data Sources used in Integra Indexes.

Data Source	Description	Data Collection	Data content	Sample size & dates	Utility for Index
**Periodic Activity Review**	Five day assessment of resource use and service organisation	Staff interviewed health facility staff and conducted observations of practice	Record of service provision for each staff member and of services provided in different locations	40 clinics at baseline (2008–9) 40 clinics at endline (2010–11**)**	Provides data on range of services in each department and room on each day of the week
**Costing study (registers)**	Micro- costing study	Record review, timesheets, and observations	Time spent on each service by staff, unit costs of all services	40 clinics at baseline (2008–9) 40 clinics at endline (2010–11)	Provides data on range of services provided by staff
**Client flow tool**	Five-day assessment of service utilisation patterns in each study clinic.	Staff gave each client entering the facility a 1pg form to carry with them until they left the clinic. Forms were filled by each provider seen.	Record of all services received by/referred for each client in every consultation over one day’s visit.	Swaziland: N = 4202 at baseline (July 2009); N = 5040 at endline (Jan 2012).Kenya: N = 4775 at baseline (July 2009); N = 5829 at endline (Jan 2012).	Data provides information on each clinic’s ability to deliver integrated services to clients (functional integration).

**The economics dataset** was derived from costing and periodic activity review tools, completed by researchers in collaboration with facility managers and staff.[24] The tools collected data on expenditures, facility characteristics, staffing and services. They included data from facility registers on services provided, as well as observations of services offered and resource use. The latter involved researchers observing staff members and facility practice over a one week period. In addition, interviews were conducted with facility staff, including completion of timesheets, to better understand how both physical infrastructure and human resources were used to provide services. The economics dataset was used to confirm the range of services available in the relevant department and facility, to estimate the average number of different HIV-RH services provided in each consultation room per day, and measure the range of services provided per staff member, and as such, provides information on the physical and human resource infrastructure that is in place and being used (structural integration).

**The client flow dataset** was derived from a five-day assessment of service utilisation patterns in each study clinic.[25] A client flow form was used to record all services received by/referred to for each client in every consultation over one day’s visit. Forms were completed by each provider seen (providers were, therefore, aware of the data being recorded). A total of 25,539 visits were tracked across 24 facilities in Kenya (10,266 in Eastern Province and 15,270 in Central Province) and 8263 visits across 8 facilities were tracked in Swaziland. Baseline and endline data only are reported in this paper: sample sizes are shown in Table 2. The dataset was used to measure which services were being offered on site; the range of services provided across days of the week; the range of services provided in single consultations; and the range provided in single visits. In this way, this data provides information on each clinic’s ability to deliver integrated services to clients (functional integration).

From these data sources, tables containing eight data points (for each attribute in Table 1) for each study clinic were constructed (S1 Table).

#### 3) Expert opinions of integration attribute weights

Given that the initial development of the index was by an internationally led study team we identified a need to further add to the development of measurement tools by using a formal process to gather local expert opinion to inform our index model. We sought the views of 23 service providers, managers and researchers from Kenya and Swaziland on the selected integration attributes in order to weigh the relative importance of attributes, allowing sensitivity within the model to different attributes of care. Participants were purposively sampled: those with knowledge of the country contexts, services being investigated and a range of the study clinics.

All participants were asked to rank the eight attributes in order of their importance to defining integrated care, using a modified Delphi technique involving ranking, discussion and re-ranking to reach consensus.[26, 27] The results of the final ranking round were used to inform the modelling process, however these findings did not change the overall substance of the models. They did not change the need for a two factor solution (see next section) but in fact worsened the fit of the models. For these reasons the expert opinion data was not used in the final modelling process for generating the Integra indexes, leaving the basis of the models objective data only.

#### 4) Generating the Integra Indexes

“Integration” is accepted as a complex phenomenon embracing multiple concepts and definitions. Integration is thus viewed as a metric whose true values cannot be directly observed [28] and the assumption is that our attributes are manifestations of this latent construct of integration.

We used latent variable models to develop models that combine information from the eight attributes of integration listed in Table 1. These models allow the combination of information from these attributes, without making any assumptions about their measurement unit, and also allow the empirical assessment of the reliability and validity of these. Results for these models are shown in Tables 3, 4 and 5.

**Table 3:** Measures of model fit for baseline models showing χ^2^ confidence interval, associated p value and Proportional Scale Reduction Criterion.

	*χ*^*2*^ 95% CI*		*p*	PSR**
Single factor model	23.442	85.117	0.001	1.001
Two factor model	-23.696	33.762	0.362	1.001

* 95% confidence interval for the difference between the observed and replicated chi square (x^2^) values—the inclusion of zero in the interval and a non-significant posterior predictive p value indicate good fit

** Proportional Scale Reduction (PSR) values close to 1 indicate model convergence

**Table 4:** Standardised factor loading scores for attributes of a single-factor model, at baseline and endline.

Integration attributes	-One Factor derived model	
	2009	2012
*Indicators of integrated service delivery (from client-flow data)*		
HIV treatment location	0.501	0.684
Range of services accessed daily	0.786	0.918
Range of services per consultation	0.983	0.988
Range of services per visit	0.982	0.993
*Indicators of structural integration (from activity reviews & register data)*		
Service availability in MCH/FP unit	-0.092	-0.106
Service availability at facility	-0.185	-0.068
Range of services per provider	-0.006	-0.049
Range of services per room	-0.151	0.126

**Table 5:** Standardised factor loading scores for the two-factor model at baseline and endline.

Integration attributes	Factor 1		Factor 2	
	Integrated service delivery: Functional Integration		Structural integration	
	2009	2012	2009	2012
*Indicators of functional integrated service delivery (from client-flow data)*				
HIV treatment location	0.489	0.672		
Range of services accessed daily	0.774	0.910		
Range of services per consultation	0.979	0.986		
Range of services per visit	0.984	0.993		
*Indicators of structural integration (from activity reviews & register data)*				
Service availability in MCH/FP unit			0.952	0.884
Service availability at facility			0.617	0.642
Range of services per provider			0.836	0.748
Range of services per room			0.795	0.736

We modelled the relationship between observed attributes of integration and latent integration using 2-parameter probit link functions for the binary and ordinal nature of the indicators.[28] In this framework the integration latent variable is influenced by all attributes, the relative contribution of each attribute to the latent summary of integration being expressed by the factor loading. For binary or ordinal attributes probit thresholds represent the level of latent integration that needs to be reached for each particular category. Given the small sample of 40 clinics, the Bayesian estimation framework was used. The Bayesian framework offers an attractive alternative to maximum likelihood estimation which may produce biased estimates in small-sample studies because of its reliance on large sample (asymptotic) theory. We employed normally distributed “non-informative priors” in an attempt to approximate maximum likelihood in our small sample.

All models were estimated using the Markov Chain Monte Carlo algorithm (four chains, 50,000 Bayes iterations) based on the Gibbs sampler, firstly on baseline data (2009) in Mplus 6.[29] Model convergence was assessed with the Proportional Scale Reduction (PSR) criterion (values close to 1 indicate model convergence) and model fit with the 95% confidence interval for the difference between the observed and replicated chi square values (the inclusion of zero in the interval and a non-significant p value indicate good fit).

## Results

### Integration measurement models

Table 3 shows criteria of model fit for the one and two-factor models using a latent variable model with non-informative priors for all parameters as an alternative to maximum likelihood estimation. Indexes of fit are shown for the baseline data only as endline data models returned very similar fit (results available from corresponding author) as well as a similar pattern of factor loadings. For the single factor model the PSR value is close to indicating convergence. However the *χ*^*2*^ 95% confidence interval for the difference between the observed and replicated *χ*^*2*^ excludes zero and the associated p value is significant, indicating poor fit. However the 2-factor model shows good fit by the same criteria.

Table 4 shows the standardised factor loadings for the model at baseline and endline and indicates a single latent dimension of integration. These loadings simply describe the relative weight or association between the attribute and the latent summary of “integration”, i.e. the strength of association. Conventionally, an absolute factor loading of 0.7 is “very satisfactory” and above 0.4 “acceptable” when assessing such a model.[30]

The factor loadings in Table 4 suggest that structural integration attributes and functional integration attributes (the ability to deliver integrated HIV-RH services) are not correlated. In other words, structural integration does not necessarily result in integrated delivery of HIV-RH services to the client. While one might expect structural integration characteristics to be correlated with (or be a pre-requisite for) integrated service delivery, in fact the findings suggest that an inverse relationship may exist. We ran further tests to assess whether the results were driven by facility size (not shown): there were no significant differences although confidence intervals were too wide to interpret.

As a result of the differences in the loadings of the attributes of *structural* and *functional* integration, as well as the poor fit of the unidimensional integration models, we estimated a two-factor data-derived (non-informative priors) model where the index was separated to create two sub-indexes. Table 5 shows the strong data-driven loadings of the attributes on each latent factor implying that these most likely reflect valid variance and not systematic error due to different data collection techniques.

Our analysis of data suggests that the structural and the functional attributes constitute two distinct and uncorrelated (orthogonal) dimensions of integration and therefore each needs to be considered separately.

The two factor model had a good fit to the data as indicated by the inclusion of zero in the *χ*^*2*^ 95% confidence interval for the difference between the observed and replicated *χ*^*2*^ and the associated non-significant p value (Table 3). Furthermore, Table 4 shows that the factor loadings remain quite consistent over the three years, indicating that the two-factor model is well replicated over time—i.e. the stability of the measurement model is good. This does not imply that the latent factors do not change over time, but that any change can be attributed to true change in integration rather than inconsistency in measuring integration.

Finally, all attributes show sizeable loadings and are directionally sensible. This situation is normally accepted as evidence of construct or internal validity and we felt confident that this was so, particularly given the better model-fit of the two-factor model and its stable factor loadings over time. As expected from the stability of factor loadings over time, the measurement equivalence of the two factor model between 2009 and 2012 was empirically confirmed (results available from corresponding author). Thus we retained two distinct latent factors: a Structural Integration Index and a Functional Integration Index.

### Clinic rankings

**Fig 1:**
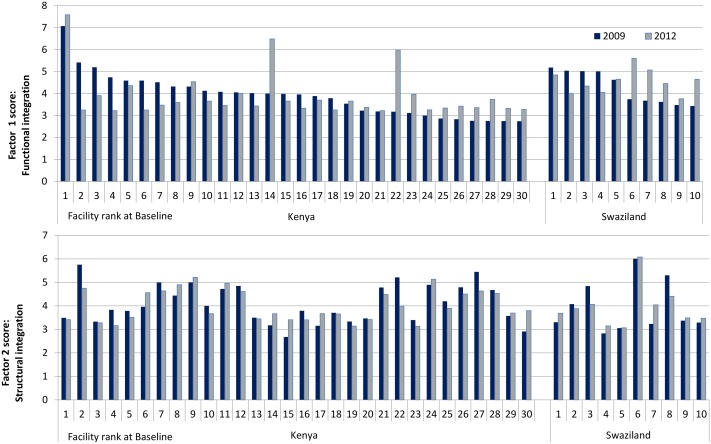
Factor scores by facility, country and time point.

To illustrate the application of the index we present Fig 1 that then applies these indexes to clinics in the study sample. Based on the two factor model latent integration scores and their corresponding standard errors, values were calculated as Bayesian plausible values [31, 32] for both factors. The stability of the factor loadings (measurement equivalence) over the three years allowed us to calculate latent scores for both waves using baseline measurement model parameters in order to obtain a fair comparison between the two time points (2009 vs 2012). Latent scores can theoretically range from minus infinity to infinity, but in practice the range is usually from -3 to 3 (transformed to 1 to 7 for graphing purposes). For the purpose of analysis, a model based latent score (Bayesian plausible value) was assigned to each clinic. The clinics were then ranked (from 1 = high, to 42 = low) with respect to the two latent dimensions of integration.

Fig 1 shows model-derived index scores for all clinics, at baseline and endline, by factor (functional/structural) and by country. In the upper panel (functional integration scores) the clinics have been ordered by descending baseline index score, from left to right for Kenya and for Swaziland. In the lower panel the clinic order is consistent with the upper panel.

Correlations between structural and functional scores were tiny in both waves (r = -0.12 (2009), -0.04 (2012)) which is to be expected as the scores are model-based from a model with two orthogonal factors.

The pattern in the upper panel (functional integration) is striking: high baseline scores have mostly diminished by endline whereas lower baseline scores have increased moderately (with two particularly notable increases in Kenya). The stability of the 2012 scores in Kenya, apart from three high scoring clinics, is quite remarkable and is likely to be a classic case of regression to the mean. The pattern is similar for Swaziland although 2012 improvements are more marked.

This paper is concerned with measurement issues rather than any program effect but, bearing in mind that latent scores were very similar for the two periods, and that we chose the baseline model to construct scores for both periods so that they were comparable, the pattern suggests an improving situation for initially low scoring clinics.

By comparison the lower panel (structural integration), when viewed in a clinic-by-clinic comparison shows a less coherent pattern (because it is ordered consistently with the upper panel, rather than by factor scores for the structural Index). If ordered in the same fashion (not shown) the differences between baseline and endline are far less marked and the pattern is less distinctive. There is still some suggestion of high scoring clinics losing score and vice versa, but this is less marked and restricted to the extremes. It seems much more likely that these smaller effects are indeed regression to the mean.

The sum of absolute differences between baseline and endline are 31.6 for the upper panel combined and 15.3 for the lower panel. A higher correlation between baseline and endline scores in the combined lower panel than in the upper panel (r = 0.83 vs 0.41 respectively) is consistent with the absolute differences.

Smaller differences for the structural integration component is perhaps not surprising since structural integration is often dictated by infrastructure, which is difficult to change. However what is of interest here is that the structural integration component has shown relatively little change between periods but the functional integration component has revealed distinctly patterned differences over time.

Whether these changes are related to program effects, external effects, or entirely regression to the mean will be subject to further analysis but the use of an objectively derived index has uncovered and quantified a pattern worthy of investigation, and has facilitated further research in a way that subjective judgements of experts, or indeed researchers, may not have done.

## Discussion

This paper addresses an important deficiency in the literature on measurement of integrated HIV-RH care by developing a tool that can be used to assess the degree of service integration. The analysis uses multiple data from a moderate number of clinics, in combination with statistical modelling, to develop a measure of service integration. This is an important advance on previous research: many previous reports simply categorize clinics as integrated or not depending on whether they received an intervention (or as self-reported by staff), but do not formally assess the extent of integration as a multi-dimensional continuum. Even where formal measurements are made previous studies have rarely included any measurement of whether clients receive integrated care (i.e. multiple HIV-RH services received by a client from one provider or in one visit).[3, 15, 16]

Two findings from our study are noteworthy. First, the two uncorrelated factors that emerged from our analysis strongly suggest that simply putting infrastructure and multi-tasking staff in place (“structural integration”) may not be sufficient to achieve integrated HIV-RH service ***receipt*** by the client (“functional integration”). This is not necessarily counter-intuitive, as it is plausible that some sites, particularly smaller ones, may have high levels of structural integration, but in practice may not be able to deliver integrated services because of barriers like vertical, duplicate reporting/recording systems, time constraints and poor staff motivation.

The emergence of two distinct dimensions illustrates the difference between the structural integration of a facility offering *potential* for integrated HIV-RH delivery and integrated HIV-RH services *actually received* by the client. This distinction is important for future outcomes assessments since it suggests that measures of physical integration and staff multi-tasking should not be used *on their own* to measure HIV-RH integration or relate integration to HIV and RH outcomes, but that a more nuanced causal pathway analysis may provide a better interpretation of results. Yet many studies do equate structural integration with integrated delivery of care and fail to explore the extent to which structural integration leads to integrated delivery, which then leads to outcomes. Our findings underline the need for a broader approach to measuring HIV-RH integration, combining a functional integration measure to better understand the link between an intervention in the area of integration and observed health outcomes.

Second, our findings show the high degree of heterogeneity across clinics in both countries, illustrating that integration is highly complex and was implemented and achieved differently in every clinic. This reinforces the need to adopt a measurement approach that allows for assessment along a continuum and is able to quantify the extent of service integration. This approach is also critical for embedded or programme-science research designs, where ensuring a standardised dose of an intervention may be influenced by a wide variety of factors beyond the control of the research study.

A number of important limitations need to be taken into account when reflecting on our findings. Costing data based on observation, self-reporting and routine service records is susceptible to a number of biases, including provider reporting bias. Client flow data, collected over one week, may not be representative of typical monthly/annual client flow (e.g. there may have been staff on training that week, national holidays, seasonal use etc.). Coordinating the same five days across facilities was logistically challenging and not always achieved. Nevertheless, clinic register data could not be used as an alternative since registers could not record how many different services each individual client received in a single consultation or visit. However, the use of multiple methods allowed for complementarity between different data sources for the indicators used, rather than relying on a single source.

Despite these limitations, the Structural and Functional Integration Indexes have useful future applications. First, they can be used to assess how clinics are changing over time relative to other clinics in terms of service integration—useful for policy or programme decision-makers interested in knowing how clinics are progressing. The general decline in clinic scores at endline (Fig 1) suggests sustaining integration over time can be challenging and further analysis is ongoing to understand the processes by which integration is successfully sustained. More work is underway to select a single or small number of indicators that could be routinely (or more easily) measured, while still providing an accurate assessment of HIV-RH integration. These indicators can be used by programme and policy decision-makers to monitor programme achievements on HIV-RH service integration in both high- and low-income settings.

Second, our Integration Indexes help identify what attributes are most closely associated with integration, or in other words what drives integration, in different contexts. This is important for policy makers and funders who wish to know where to channel resources. Many impact studies of integration focus on the impact of an intervention on observed outcomes but fail to account for the drivers of this impact. [33,34] For example, observed improvements in uptake of services following integration could be the result of better supported, more motivated staff at intervention sites, rather than the mere presence of an additional service. Consequences of this for funding are important since it may be that large-scale investment in structural components (e.g. roll-out of drugs or test-kits on site; clinical training of staff) may not result in sustained improvements in health outcomes unless equally large investments are made in supporting the workforce (e.g. through mentorship, quality supervision). A growing literature on the importance of supporting the “software” (people) within health systems and services, beyond narrow clinical training, is important here. [35, 36] To further our understanding of the drivers of integration, we are conducting further analysis of the individual attributes to determine whether a sequencing of inputs can be identified (e.g. do you need physical and human resource integration in place before you get availability of services within an MCH unit and what enables this to lead to *delivery* of integrated care?) [37,38]. Third, the Indexes can enable researchers to improve the assessment of the attribution of a particular health or service outcome to service integration.[3] For example, within the Integra Initiative we are using the Indexes to provide a sophisticated measure to assess a dose-response relationship between women’s cumulative exposure to integrated services and study outcomes, including HIV-testing, condom use and technical quality of care.[39] Where positive impacts are correlated with functional integration we are conducting further analysis to determine drivers of functional integration.

Furthermore, beyond SRH/HIV integration, the principles of the need to measure both a dimension of “structural” service integration and “functional” delivery of integrated care may be applicable for other service-integration packages, though further application is needed to test this.

To conclude, the Integra Indexes strongly suggest that “integration” exists in two forms that are distinct but easily confused. Functional integration is linked to actual receipt of multiple services at one time and place and may be unrelated to structural integration where different services are “available”, but not necessarily provided in an integrated form. Furthermore, our findings have important implications for research on integrated HIV-RH services since they underline the importance of 1) having a measure to quantify the degree of service integration; 2) assessing both structural integration (physical and human resources) and functional integration (delivery of integrated services to a client) in order to determine the ‘attainment’ of integrated services. We conclude, that the Integra Indexes are a useful conceptual and methodological contribution to measuring HIV-RH integration and enabling the attribution of particular health/service outcomes to integration—achievements which have proven elusive to date.

## Supporting Information

S1 Table Descriptive Data for Eight Variables in the Integra Index Models, by clinic at baseline and endline. (DOCX)

S2 Table Supporting data for Fig 1: baseline and endline clinic scores, by country. (XLS)
