# Supplementary material for: Innovation in Evaluating the Impact of Integrated Service-Delivery: The Integra Indexes of HIV and Reproductive Health Integration
Source: PLoS One. 2016 Jan 22;11(1):e0146694. doi: 10.1371/journal.pone.0146694 (PMC4723242; doi:10.1371/journal.pone.0146694)
Supplement: S1 Table — (DOCX) [file pone.0146694.s001.docx]

**Supplementary Data Table 1:**

**Descriptive Data for Eight Variables in the Integra Index Models, by clinic at baseline and endline**

| **Facility rank number at baseline** | **HIV treatment location ˟̂** | | **% of days in the week on which any RH AND**  **HIVˠ service accessed** | | **% Clients receiving any RH AND any HIV service in one consultation** | | **% Clients receiving any RH AND any HIV service in one visit** | | **% HIV services available at MCH/FP unit** | | **% HIV and RH Services available at facility** | | **% HIV and RH services provided per clinical staff member in MCH/FP unit˄** | | **% HIV and RH services provided per consultation room in MCH/FP unit˄** | |
| --- | --- | --- | --- | --- | --- | --- | --- | --- | --- | --- | --- | --- | --- | --- | --- | --- |
|  | **2009** | **2012** | **2009** | **2012** | **2009** | **2012** | **2009** | **2012** | **2009** | **2012** | **2009** | **2012** | **2009** | **2012** | **2009** | **2012** |
| **KENYA** | | | | | | | | | | | | | | | | |
| 1 | 0.04 | 0.17 | 100 | 80 | 71.43 | 71.26 | 71.43 | 71.26 | 20 | 40 | 63 | 75 | 20 | 40 | 7 | 33 |
| 2 | 0.035 | 0 | 100 | 0 | 44.16 | 0 | 44.16 | 0 | 40 | 40 | 63 | 75 | 40 | 40 | 10 | 30 |
| 3 | 0.042 | 0.02 | 100 | 100 | 37.56 | 10.3 | 42.25 | 10.3 | 60 | 60 | 100 | 100 | 60 | 60 | 15 | 15 |
| 4 | 0.31 | 0 | 100 | 20 | 30.14 | 0 | 35.62 | 0 | 60 | 60 | 100 | 100 | 60 | 60 | 60 | 20 |
| 5 | 0 | 0 | 60 | 60 | 31.37 | 18.6 | 31.37 | 18.60 | 60 | 60 | 75 | 75 | 60 | 47 | 30 | 33 |
| 6 | 0.01 | 0.01 | 100 | 40 | 30.16 | 0 | 30.95 | 1.00 | 40 | 40 | 100 | 88 | 40 | 40 | 40 | 13 |
| 7 | 0 | 0 | 60 | 60 | 26.32 | 3.66 | 31.58 | 3.66 | 80 | 80 | 88 | 88 | 46 | 55 | 40 | 50 |
| 8 | 0.07 | 0 | 80 | 40 | 25.58 | 0 | 25.58 | 11.76 | 20 | 20 | 75 | 88 | 20 | 13 | 20 | 7 |
| 9 | 0.04 | 0.05 | 100 | 100 | 25.71 | 20.27 | 27.14 | 21.62 | 60 | 40 | 88 | 88 | 60 | 40 | 40 | 40 |
| 10 | 0.012 | 0.01 | 100 | 80 | 18.02 | 5.85 | 24.42 | 6.83 | 60 | 60 | 88 | 88 | 60 | 60 | 12 | 20 |
| 11 | 0 | 0.03 | 100 | 20 | 17.86 | 3.23 | 25 | 3.23 | 20 | 20 | 50 | 63 | 20 | 20 | 20 | 20 |
| 12 | 0 | 0.04 | 100 | 60 | 21.92 | 10.71 | 21.92 | 14.29 | 20 | 20 | 50 | 75 | 20 | 15 | 20 | 20 |
| 13 | 0.01 | 0 | 100 | 40 | 20.16 | 3.03 | 20.97 | 3.03 | 40 | 40 | 63 | 75 | 40 | 39 | 10 | 20 |
| 14 | 0.00 | 0.10 | 100 | 80 | 14.29 | 53.33 | 24.29 | 53.33 | 20 | 40 | 38 | 75 | 20 | 36 | 7 | 20 |
| 15 | 0.58 | 0.03 | 80 | 75 | 14.06 | 6.25 | 23.44 | 6.25 | 40 | 20 | 75 | 63 | 40 | 20 | 13 | 10 |
| 16 | 0.02 | 0 | 60 | 20 | 20.45 | 0 | 20.45 | 2.13 | 0 | 20 | 38 | 75 | 0 | 20 | 0 | 20 |
| 17 | 0.11 | 0.06 | 100 | 80 | 16.06 | 6.78 | 18.98 | 8.47 | 20 | 40 | 88 | 100 | 20 | 40 | 4 | 8 |
| 18 | 0 | 0 | 80 | 0 | 16.9 | 0 | 16.9 | 0 | 20 | 20 | 50 | 63 | 20 | 20 | 20 | 20 |
| 19 | 0 | 0.05 | 40 | 40 | 0 | 6.33 | 20 | 6.33 | 80 | 80 | 88 | 88 | 48 | 53 | 40 | 24 |
| 20 | 0 | 0.01 | 20 | 80 | 0 | 1.55 | 11.11 | 3.10 | 80 | 80 | 88 | 88 | 40 | 40 | 45 | 52 |
| 21 | 0.27 | 0 | 100 | 0 | 0.97 | 0 | 9.18 | 0 | 40 | 40 | 88 | 75 | 40 | 40 | 40 | 13 |
| 22 | 0 | 0 | 20 | 100 | 0 | 44.76 | 11.76 | 44.76 | 80 | 80 | 88 | 88 | 52 | 60 | 47 | 66 |
| 23 | 0.233 | 0.01 | 100 | 100 | 0 | 10 | 8.3 | 14.36 | 60 | 60 | 100 | 100 | 34 | 60 | 20 | 40 |
| 24 | 0 | 0 | 20 | 20 | 0 | 0 | 4.55 | 0 | 80 | 80 | 88 | 88 | 43 | 50 | 60 | 40 |
| 25 | 0 | 0.01 | 20 | 40 | 1.85 | 2.00 | 1.85 | 2.00 | 40 | 60 | 88 | 88 | 40 | 53 | 20 | 30 |
| 26 | 0.01 | 0.01 | 60 | 100 | 0.43 | 0.81 | 1.28 | 3.23 | 40 | 40 | 100 | 100 | 40 | 33 | 13 | 8 |
| 27 | 0 | 0 | 20 | 20 | 0 | 0 | 0 | 3.64 | 40 | 20 | 75 | 63 | 40 | 15 | 20 | 10 |
| 28 | 0 | 0 | 0 | 20 | 0 | 8.33 | 0 | 8.33 | 20 | 20 | 63 | 75 | 20 | 20 | 20 | 20 |
| 29 | 0 | 0.04 | 0 | 20 | 0 | 0 | 0 | 1.67 | 80 | 80 | 88 | 88 | 65 | 40 | 60 | 51 |
| 30 | 0 | 0 | 0 | 0 | 0 | 0 | 0 | 0 | 20 | 20 | 50 | 63 | 20 | 20 | 20 | 20 |
| **SWAZILAND** | | | | | | | | | | | | | | | | |
| 1 | 0.63 | 0.98 | 100 | 100 | 35.90 | 24.47 | 42.74 | 27.66 | 20 | 20 | 88 | 75 | 10 | 20 | 20 | 20 |
| 2 | 0.07 | 0.67 | 100 | 100 | 38.24 | 10.34 | 39.22 | 13.36 | 40 | 20 | 88 | 88 | 20 | 20 | 10 | 15 |
| 3 | 0.34 | 0.97 | 100 | 100 | 35.06 | 16.07 | 38.31 | 19.64 | 40 | 60 | 100 | 100 | 7 | 17 | 10 | 16 |
| 4 | 0.06 | 0.67 | 100 | 80 | 37.30 | 11.31 | 37.70 | 14.48 | 60 | 60 | 75 | 75 | 60 | 28 | 60 | 53 |
| 5 | 0.26 | 1.05 | 100 | 100 | 29.04 | 21.12 | 33.49 | 24.84 | 80 | 80 | 88 | 88 | 80 | 80 | 60 | 80 |
| 6 | 0.13 | 1.24 | 100 | 100 | 13.44 | 37.17 | 17.39 | 40.13 | 20 | 20 | 88 | 88 | 7 | 6 | 5 | 7 |
| 7 | 0.13 | 0.19 | 80 | 100 | 12.50 | 27.12 | 15.18 | 32.77 | 20 | 20 | 75 | 100 | 8 | 5 | 7 | 4 |
| 8 | 0.12 | 0.27 | 100 | 100 | 10.75 | 18.93 | 15.77 | 19.95 | 60 | 60 | 100 | 100 | 51 | 11 | 24 | 9 |
| 9 | 0.30 | 0.35 | 100 | 100 | 9.57 | 7.42 | 12.87 | 8.98 | 80 | 60 | 88 | 75 | 14 | 7 | 15 | 20 |
| 10 | 0.03 | 0.09 | 100 | 100 | 9.92 | 22.94 | 13.22 | 23.24 | 20 | 60 | 50 | 75 | 16 | 7 | 20 | 12 |
| Mean | **0.10** | **0.18** | **75** | **61.88** | **17.93** | **12.10** | **21.76** | **13.71** | **44.5** | **46** | **78.4** | **83.05** | **35.03** | **33.75** | **25.10** | **25.23** |
| Median | **0.03** | **0.02** | **100** | **77.5** | **16.5** | **6.6** | **20.7** | **8.4** | **40** | **40** | **88** | **88** | **40** | **37.5** | **20** | **20** |
| SD | **0.16** | **0.34** | **35.30** | **36.68** | **16.15** | **16.02** | **15.34** | **16.08** | **23.75** | **22.28** | **18.30** | **11.87** | **19.61** | **18.99** | **17.85** | **17.38** |

˟̂ HIV location: Referral for ART mean score: 0=Received no ART ("HIV care") and not referred for ART; 1=Referred for ART but not received during visit; 2=Received ART during visit, either as 1 service only, or as additional service but with a different provider; 3=Received ART in addition to an SRH service (FP/ANC/PNC/STI) and with the same provider.

ˠ RH service defined as any of: 1) FP; 2) ANC; 3) PNC; 4) cervical cancer screening

HIV service defined as any of: 1) Antiretroviral therapy (ART); 2) Cervical cancer screening; 3) CD4 count services; 4) HIV/AIDS testing services; 5) STI treatment

˄Annual Average
